# Design and engineering of water-soluble light-harvesting protein maquettes†
†Electronic supplementary information (ESI) available: Experimental methods and supplementary Fig. S1–10. See DOI: 10.1039/c6sc02417c



**DOI:** 10.1039/c6sc02417c

**Published:** 2016-08-17

**Authors:** Goutham Kodali, Joshua A. Mancini, Lee A. Solomon, Tatiana V. Episova, Nicholas Roach, Christopher J. Hobbs, Pawel Wagner, Olga A. Mass, Kunche Aravindu, Jonathan E. Barnsley, Keith C. Gordon, David L. Officer, P. Leslie Dutton, Christopher C. Moser

**Affiliations:** a The Johnson Research Foundation and Department of Biochemistry and Biophysics , University of Pennsylvania , Philadelphia , PA 10104 , USA . Email: moserc@mail.med.upenn.edu; b The ARC Centre of Excellence for Electromaterials Science and the Intelligent Polymer Research Institute , University of Wollongong , NSW 2522 , Australia; c N Carolina State University , Department of Chemistry , Raleigh , NC 27695 , USA; d University of Otago , Department of Chemistry , Dunedin 9016 , New Zealand

## Abstract

Natural selection in photosynthesis has engineered tetrapyrrole based, nanometer scale, light harvesting and energy capture in light-induced charge separation. By designing and creating nanometer scale artificial light harvesting and charge separating proteins, we have the opportunity to reengineer and overcome the limitations of natural selection to extend energy capture to new wavelengths and to tailor efficient systems that better meet human as opposed to cellular energetic needs. While tetrapyrrole cofactor incorporation in natural proteins is complex and often assisted by accessory proteins for cofactor transport and insertion, artificial protein functionalization relies on a practical understanding of the basic physical chemistry of protein and cofactors that drive nanometer scale self-assembly. Patterning and balancing of hydrophobic and hydrophilic tetrapyrrole substituents is critical to avoid natural or synthetic porphyrin and chlorin aggregation in aqueous media and speed cofactor partitioning into the non-polar core of a man-made water soluble protein designed according to elementary first principles of protein folding. This partitioning is followed by site-specific anchoring of tetrapyrroles to histidine ligands strategically placed for design control of rates and efficiencies of light energy and electron transfer while orienting at least one polar group towards the aqueous phase.

## Abstract 2

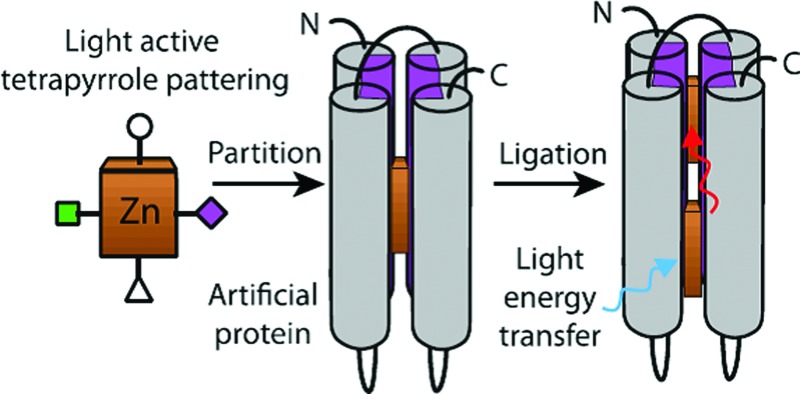
Design of nanometer scale artificial light harvesting and charge separating proteins enables reengineering to overcome the limitations of natural selection for efficient systems that better meet human energetic needs.

## Introduction

Natural photosynthetic systems embed nanometer scale light-harvesting protein complexes with tetrapyrrole and carotenoid cofactors to capture and direct light ranging from ultraviolet to near infrared. The particular wavelengths absorbed and emitted by these tetrapyrroles are tuned as they are chemically modified by peripheral substituents and as they interact with each other and the protein environment through metal ligation, hydrophobic partitioning, hydrogen bonding and aromatic pi-stacking.^1^

There is an active effort to understand how to construct efficient artificial systems that can replicate and enhance the light harvesting capabilities of natural photosynthetic systems in order to form high-energy products for a sustainable future.^2^ To this end, researchers have modified the cofactor environment in natural proteins through mutation,^3^ replaced natural cofactors with alternative cofactors in natural proteins,^4,5^ and constructed novel artificial proteins with both natural and synthetic cofactors.^6–9^ There is also growing interest in developing water-soluble light-harvesting proteins, as an alternative to the predominantly membrane bound forms found in nature, for novel applications both *in vitro* and *in vivo* to capture solar energy. Potential applications range from development of light-harvesting materials at the nano to meso scale (such as dye sensitized solar cells), to engineered coupling of solar energy to specific metabolic pathways for *in vivo* production of fuels and reagents, to therapeutic treatments such as photodynamic therapy.

**Fig. 1:**
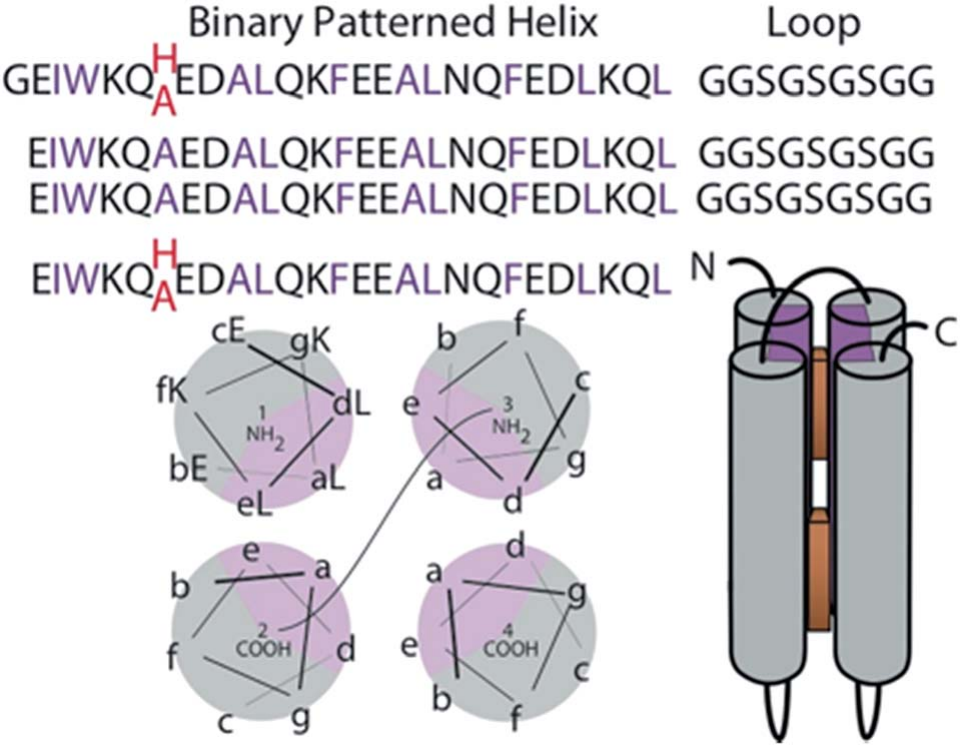
Sequence of single chain four-helix maquette for light harvesting. Binary patterning of polar (black) and hydrophobic residues (purple) drives helical bundle association in aqueous solution (bottom). The sequence with histidine at position 7 and 112 ligates Zn tetrapyrroles (brown at right), while the control sequence with alanines is histidine free.

This work exploits novel tetrapyrrole binding four-helix proteins with no sequence similarity to natural proteins; these robust and adaptable proteins are called maquettes^10^ (Fig. 1). As completely artificial proteins, they have not been naturally selected to interact with the pigment transport and insertion proteins naturally evolved to manage cofactor insertion in the living cell. Instead they are engineered for appropriate control of the physical chemical properties of cofactor and protein to support efficient self-assembly. Experience has shown that maquettes self-assemble a wide range of tetrapyrrole cofactors both on the bench top and during expression within the cell.^8,9,11–13^ While planning the synthesis of an extended range of cofactors for novel spectroscopic properties and light-harvesting application, we need to avoid compromising the speed and affinity of self-assembly, aiming to maintain nM affinities and seconds timescale binding. This means we need to avoid a common issue of tetrapyrrole cofactor aggregation in aqueous environments,^14^ but still facilitate entry and anchoring of cofactors in the hydrophobic protein interior.

Here we systematically explore the binding of Zn tetrapyrroles with different patterns of polar and non-polar substituents to four-alpha-helix maquettes engineered with either two widely spaced histidines for independent Zn metal ligation or lacking ligating histidines altogether. We use phenyl rings with and without sulphonato or carboxy functional groups to vary tetrapyrrole polarity, obtaining dissociation constants (*K*_d_ values) through singular value decomposition (SVD) analysis of visible spectra during binding titrations. We find that a balance of polar and non-polar substituents on porphyrins is key to fast and efficient binding for both porphyrins and chlorins. Amphiphilic tetrapyrroles, with one non-polar end and one polar end, allow for efficient hydrophobic partitioning into the interior of the protein while the hydrophilic part remains exposed to the aqueous portion of the protein stabilizing the complex by polar interactions. Partitioning facilitates histidine ligation to central Zn, which in turn thermally stabilizes the protein–tetrapyrrole complex by ∼3.5 kcal mol^–1^.

## Results

### Maquette design

Construction of elementary 4 α-helix bundle maquette protein frames in which positioning tetrapyrroles follows first principles of protein folding.^15^ The hydrogen bonding of the amide backbone completes two turns of the helix every 7 amino acids. Thus arranging the amino acids of high alpha helical propensity in the heptad pattern of the polar (P) and hydrophobic (H) residue PHHPPHP results in a helix with a conspicuous polar and non-polar face (gray and purple in Fig. 1). In an aqueous environment, the hydrophobic faces are spontaneously buried into the bundle core. Exterior polar residues are diversified to aid NMR structural resolution. This single chain design has been discussed previously.^7^ Two histidines are placed at interior positions 7 and 112 at opposite ends of the bundle to provide possible ligands for the Zn metal of added tetrapyrroles (Fig. 1). Alternatively, these histidines are replaced with alanines to create a ligand-free control sequence.

### Porphyrin design and binding

**Fig. 2:**
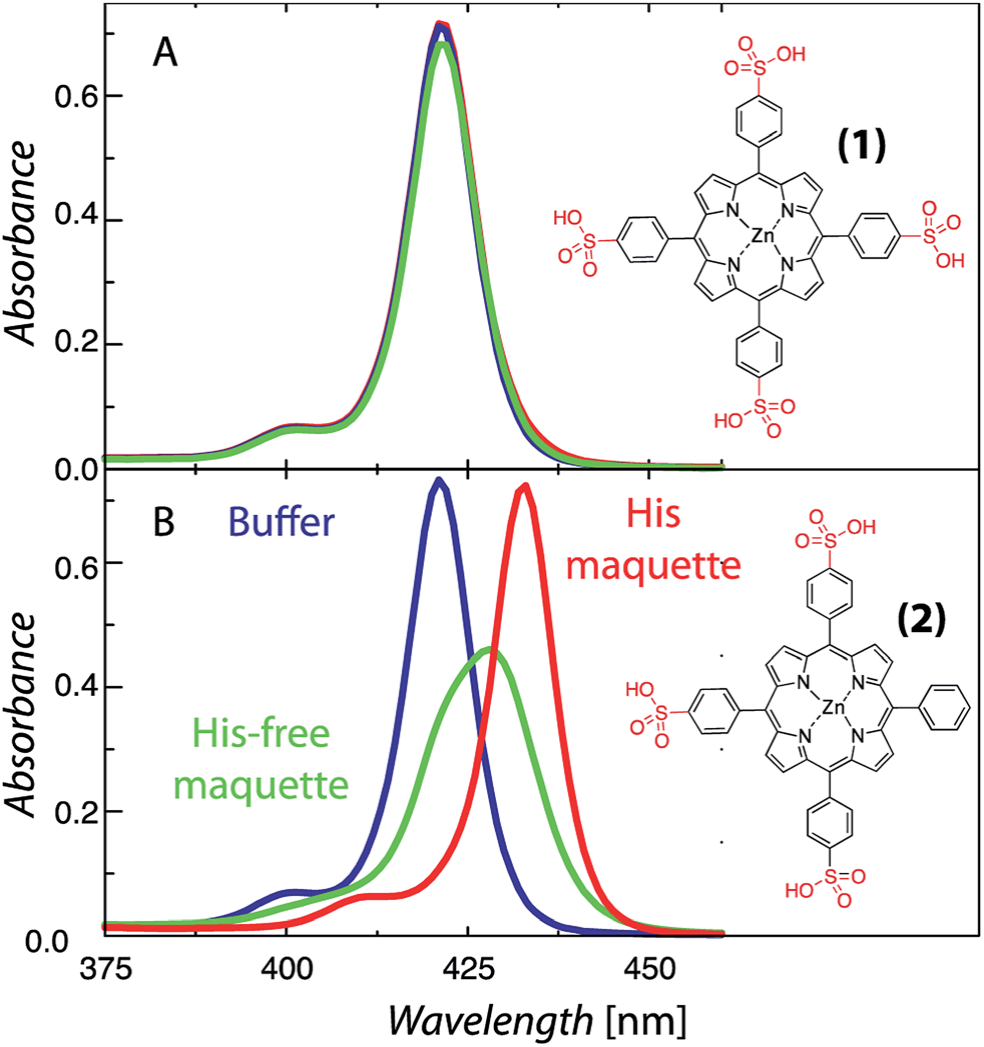
Absorption spectra demonstrating that hydrophobic partitioning is key for maquette binding. Blue, green and red spectra correspond to no maquette, His-free maquette or His-containing maquette, respectively. Panel (A) and (B) show spectra for 4 μM porphyrins (**1**) or (**2**) respectively with 2 μM maquette, when present.

The structural requirements for tetrapyrrole self-assembly in aqueous solution were first tested with Zn tetraphenyl porphyrins. The solubility in polar and non-polar environments was adjusted through combinations of hydrophobic and hydrophilic groups at the *meso* positions. Zn tetrapyrrole pigments discussed in this text are abbreviated with bold numbers, with full chemical names given in the Experimental section and structures illustrated in the figures. When all four positions are substituted with sulfonatophenyl groups, as in Zn(ii) 5,10,15,20-tetra(4-sulphonatophenyl)porphyrin (**1**), the Zn tetrapyrrole becomes highly water-soluble. Fig. 2A demonstrates that the spectrum remains unchanged when either maquette with histidines or without histidines is added, indicating that the tetrapyrrole does not associate with either maquette. However, when one sulfonatophenyl group is replaced with the non-polar phenyl group in Zn(ii) 5,10,15-tri(4-sulphonatophenyl)-5-phenylporphyrin (**2**) (Fig. 2B), the His-free control maquette shows broadening and noticeable red-shifting of the Soret absorbance compared to the tetrapyrrole in buffer, indicating partial association of the tetrapyrrole with the maquette, presumably through hydrophobic partitioning and partial burial of the porphyrin in the maquette interior. The His containing maquette generates a larger, 10 nm red shift (432 nm *vs.* 422 nm) and maintains the narrow Soret bandwidth, indicative of histidine ligation. Thus, one non-polar edge of a Zn porphyrin was sufficient to provide facile binding.

**Fig. 3:**
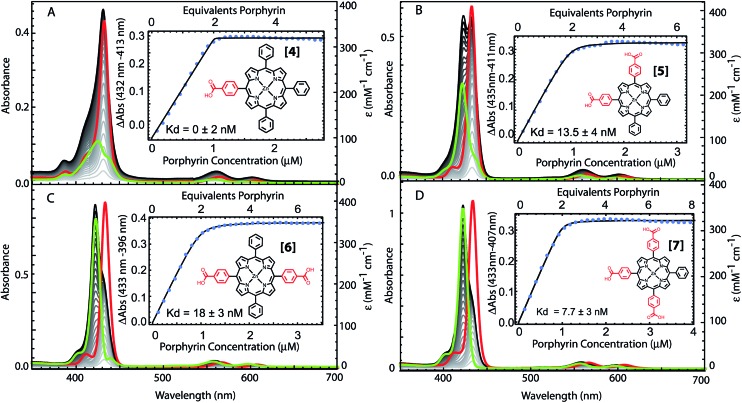
The absorption spectra and binding curves for amphiphilic Zn porphyrins with various polar carboxyphenyl substituents (red) showing binding to maquettes. Gray curves: successive absorbance spectra of titration. Red and green curves: bound and unbound tetrapyrrole spectra from SVD with extinction coefficients on right scale.

**Fig. 4:**
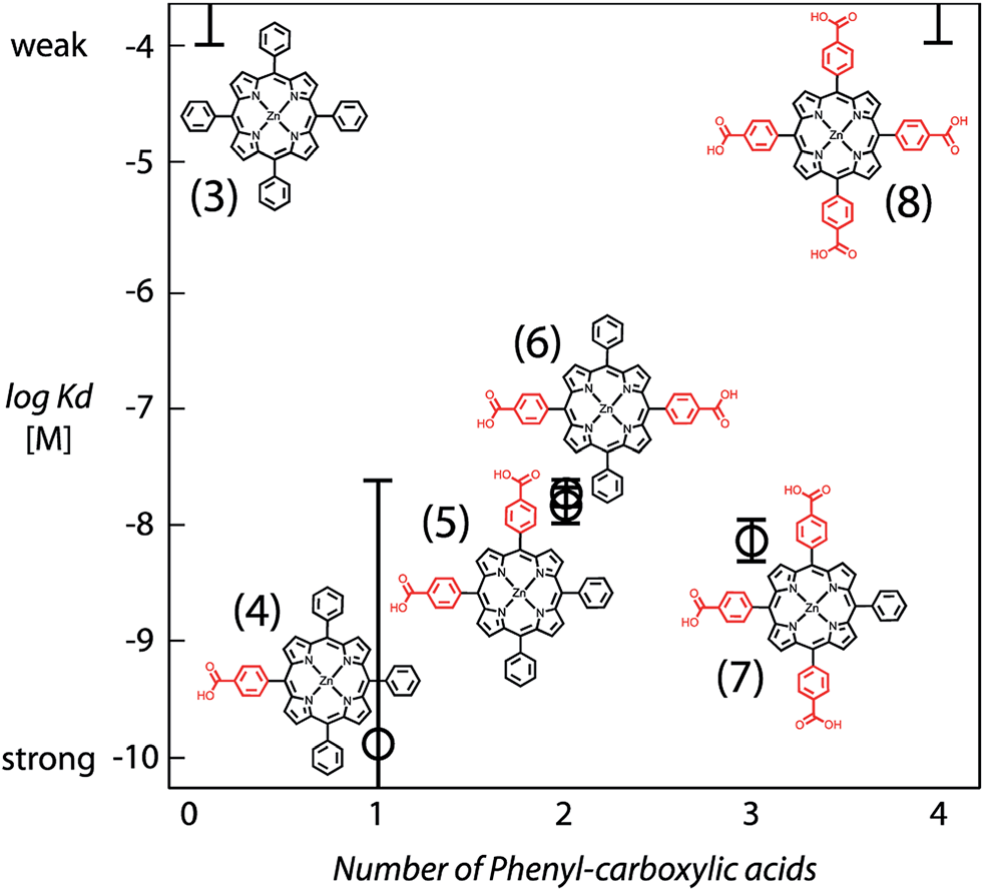
Comparative binding affinity of amphiphilic Zn porphyrins to maquettes. Polar groups are highlighted in red. Binding of (**3**) and (**8**) are too weak to measure accurately.

To determine the acceptable range and pattern of hydrophobic *vs.* hydrophilic perimeters for Zn porphyrins, we synthesized all permutations of phenyl and carboxyphenyl tetra *meso* substituents (Fig. 3 and 4). The visible spectral changes that occurred as these porphyrins were titrated into a His maquette solution were fitted to a simple binding model with a single dissociation constant (*K*_d_) for each His site using singular value decomposition.^16^ Porphyrins with either all-hydrophobic substituents, Zn(ii) 5,10,15,20-tetraphenylporphyrin (**3**), or all-hydrophilic substituents, Zn(ii) 5,10,15,20-tetra(4-carboxyphenyl)porphyrin (**8**), showed no detectable spectral shift, signifying that binding was weaker than 100 μM. Clear spectral shifts were observed for the mono-phenylcarboxylic acid (**4**), the *syn*-diphenylcarboxylic acid (**5**), the *anti*-diphenylcarboxylic acid (**6**) and the tri-phenylcarboxylic acid (**7**) with fitted *K*_d_ values of 0.14 ± 2 nM, 18 ± 3.0 nM, 14 ± 4 nM and 8 ± 3 nM, respectively. At least one hydrophobic and one hydrophilic group substitution is necessary for porphyrin ligating to the maquettes with nanomolar affinities. Mono-carboxyphenylporphyrin (**4**) affinity is estimated at about two orders of magnitude tighter than both di-carboxyphenyl porphyrins (**5**) and (**6**) and the tricarboxyphenol porphyrin (**7**).

Resonance Raman changes of the bound tetrapyrroles show changes in “core size” vibrations^17^ upon addition of maquette (Fig. S2–S5†) consistent with binding and histidine ligation. The most drastic spectroscopic change occurred for data collected at 448 nm. In the region of band ν_4_ and ν_29_ (1340–1360 cm^–1^), the lowest energy ν_4_ decreases in intensity (over 40%) relative to ν_29_. Frequency changes with regards to ν_4_ are well documented as a way to detect environmental changes close to the porphyrin core. At 1421 cm^–1^, there is a considerable decrease in intensity of the ν_3_ band, whilst ν_12_ at 1296 cm^–1^ completely disappears. At the lower energy region, there is also a red shift of the 1016 cm^–1^ band (ν_15_) to 1003 cm^–1^. Metalloporphyrin vibrational modes such as these are generally sensitive to planarity, metal oxidation state/spin or the presence of an axillary moiety, and are expected to be affected by Zn porphyrin binding to a maquette histidine.

**Fig. 5:**
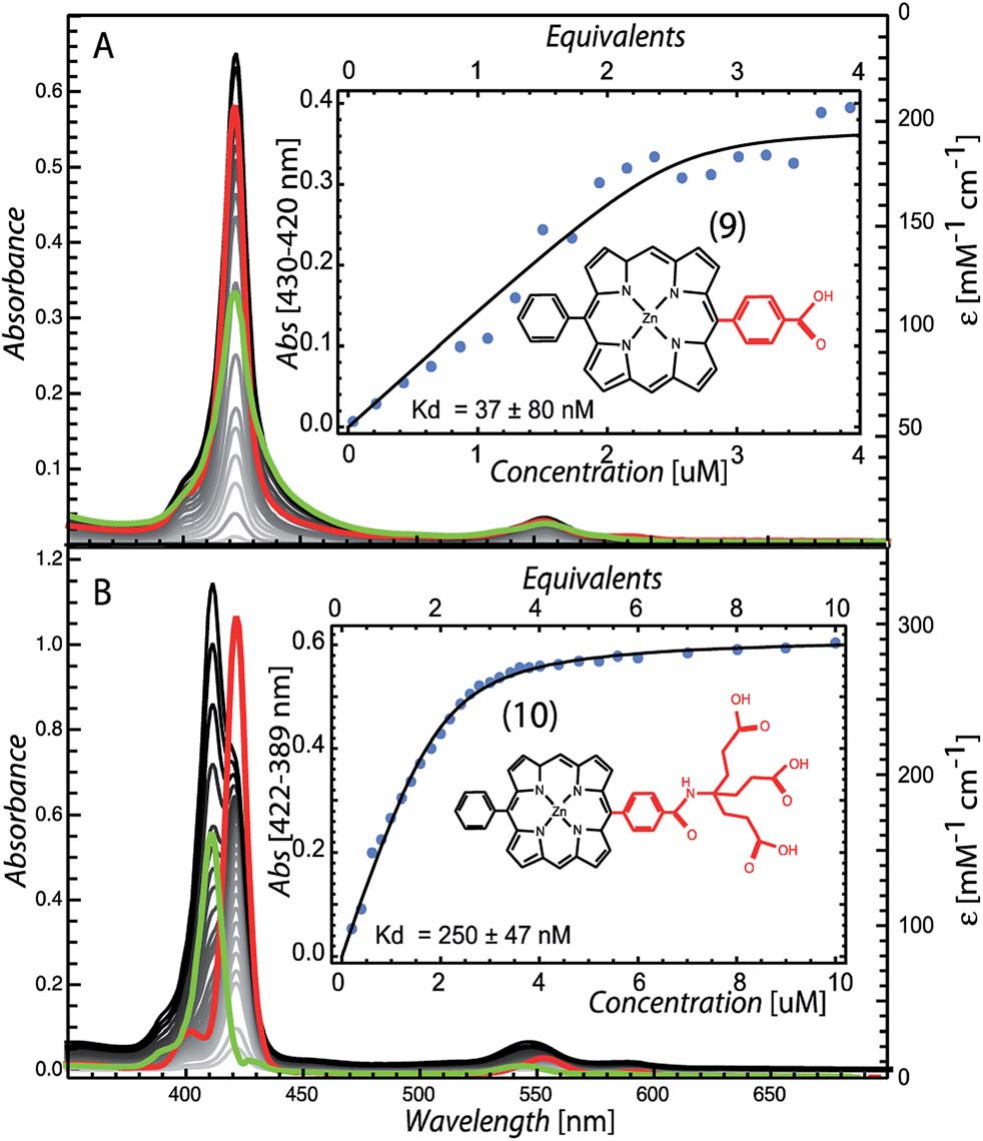
Maquette binding of amphiphilic 5,15-substituted Zn porphyrins with relatively short (**9**) and extended (**10**) polar groups.

To determine if the pattern of enhanced binding affinity with amphiphilic character of 5,10,15,20-tetrasubstituted porphyrins also applies to simpler 5,15-disubstituted porphyrins, we characterized the binding of the relatively simple carboxyphenyl construct (**9**) (Fig. 5). Although this amphiphilic Zn porphyrin apparently displays desirable <100 nM affinity, stopped flow mixing shows that binding equilibrium occurs slowly and reaches completion in 20 minutes (Fig. S1†). Fig. 5 also shows that the unbound species has a red shifted Soret typical of multimerized tetrapyrrole, which complicates accurate determination of the binding affinity. Strengthening the polar character of the 15 substituent by replacing the single carboxylic group with a first generation Newkome dendrimer made up of three carboxylic acid groups (**10**), substantially increased the water solubility of the porphyrin, eliminated pigment multimerization and permitted binding on a few ms timescale (Fig. S1†).

### Chlorin design and binding

**Fig. 6:**
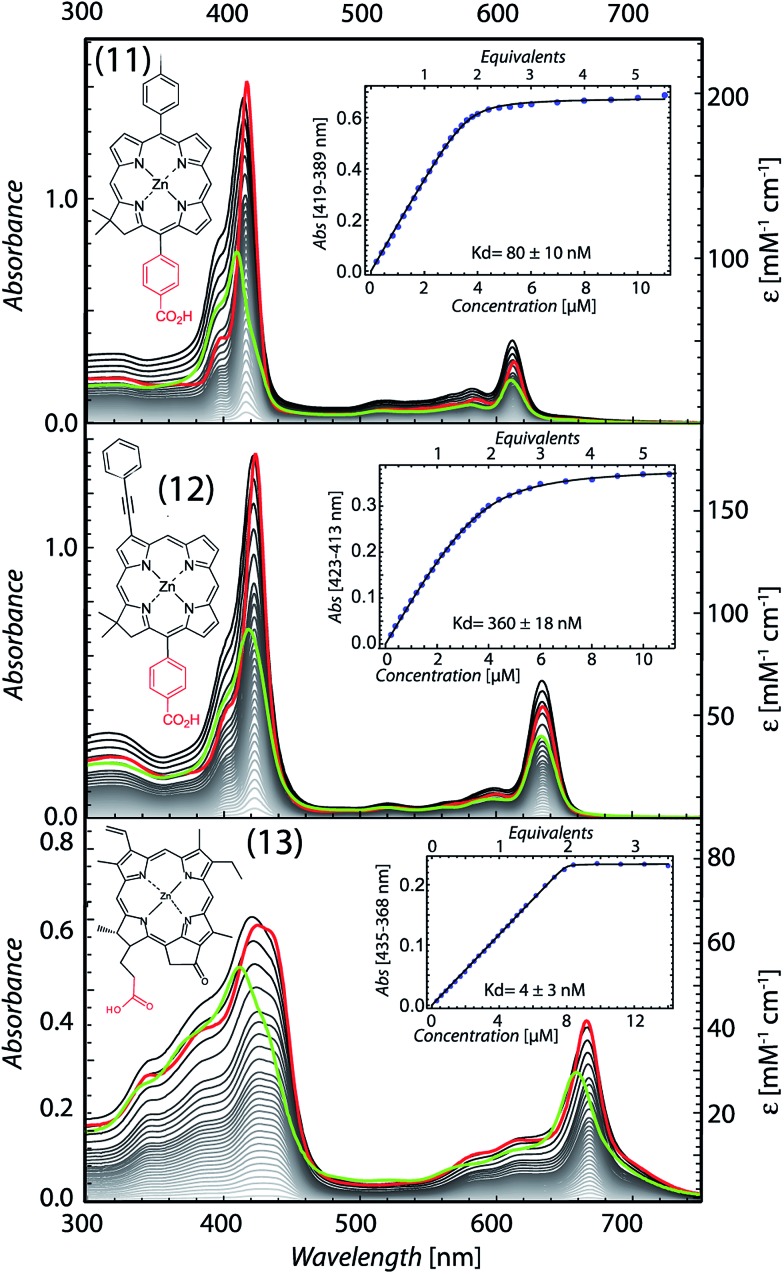
Chlorins bind with high affinity. Chlorin induced bandshifts of (**11**), (**12**) and (**13**) upon binding to maquettes were used to estimate binding stoichiometry and *K*_d_ values. Red traces show the chlorin bound to maquette absorbance spectra while green traces show the chlorin unbound absorbance spectra resolved using SVD analysis. Isosbestic points, from which the maximal difference between bound and unbound chlorin absorbance could be obtained, were determined from this SVD and plotted as insets.

Chlorin absorption bands generally have significant red shifts compared to porphyrins and so provide a complementary spectral region of light harvesting compared to porphyrins. To determine if the pattern of enhanced binding affinity of Zn porphyrins with amphiphilic character also applies to chlorins, we designed and synthesized chlorins with the analogous amphiphilic profile of 5,15-substituted tetrapyrroles. In (**11**), a *p*-tolyl substituent at the 5-position provides a hydrophobic group and a carboxyphenyl group at the opposite, 15-position provides a polar group for water solubility. These chlorins also included a dimethyl group on the pyrroline ring to stabilize the macrocycle against adventitious dehydrogenation.^18^ This chlorin is a close analogue of porphyrin (**9**). A shift of the chlorin Soret band from 411 to 418 nm and increases in extinction coefficients in both the Soret and Q bands correlate with histidine ligation on maquette binding (top Fig. 6). SVD analysis of the spectral cofactor titration resolves bound and unbound cofactor spectra. This allows us to select a wavelength absorption difference pair of 419 and 389 nm that is isosbestic for unbound cofactor, allowing a simpler view of the extent and stoichiometry of binding in the graphical insert, for a *K*_d_ of 80 ± 10 nM.

**Fig. 7:**
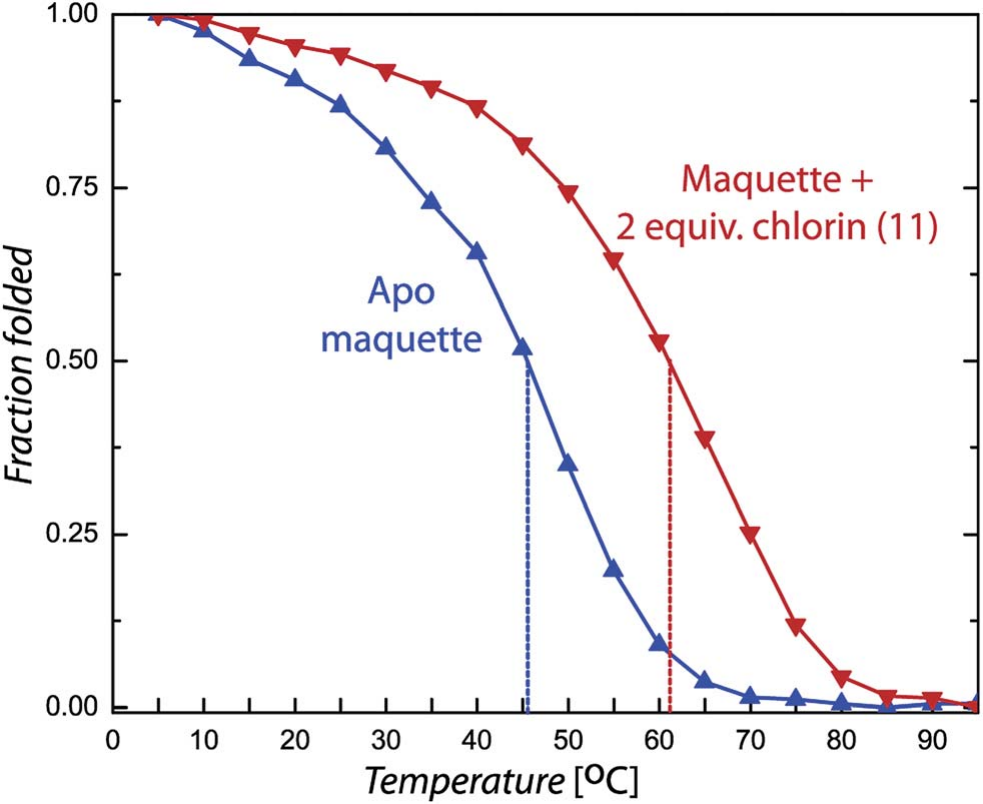
Thermal stability of the maquette increased upon tetrapyrrole binding. Circular dichroism at 222 nm monitors alpha helical folding for 2 μM maquette without (blue) or with (red) 2 equivalents of (**11**) as a function of temperature.

Chlorin (**11**) binding also stabilizes the helical structure of the maquette as seen by circular dichroism spectroscopy (Fig. 7). Thermal mid point transition of α-helical structure (*T*_m_) increases from 46 to 61 °C upon chlorin binding with a sigmoidal behavior suggesting cooperative melting. This *T*_m_ shift corresponds to 6.8 kcal mol^–1^ of structure stabilization. Stopped-flow mixing shows that the chlorin (**11**) binds much faster than the similarly substituted porphyrin (**9**).

We exploited the ability to manipulate tetrapyrrole substituents to shift light harvesting Q_y_ absorption bands to the red, while maintaining a hydrophobic/hydrophilic balance, by replacing the 5-*p*-tolyl group of (**11**) with a 3-phenylethynyl group (**12**). SVD analysis clarified binding induced bandshifts, shown together with a graphic representation of the bound pigment titration at the 423 minus 313 nm isosbestic for unbound pigment in Fig. 6 (middle). While changing the phenyl group at 5-position to the bulkier phenylethynyl group at the 3-position lowers the affinity 4.5 fold (*K*_d_ of 360 ± 18 nM), binding is still strong enough to drive the self-assembly of the protein tetrapyrrole complex.

Pheophorbide a is a popular, comparatively soluble chlorin derivative of chlorophyll in which the hydrophobic phytol tail is cleaved to leave a propionic acid. The extra oxophorbine ring (compared to the synthetic chlorins) introduces an asymmetry that makes them less prone to aggregation. We replaced the central Mg with Zn to make Zn pheophorbide a (**13**). Like the synthetic chlorins (**11**) and (**12**), (**13**) has a charged group on one edge of the tetrapyrrole, but also includes some uncharged but polar groups on this same edge. SVD binding analysis and 435 minus 368 nm difference absorption to track binding is shown in Fig. 6 (bottom), with a tight *K*_d_ of 4 ± 3 nM.

### Energy transfer

**Fig. 8:**
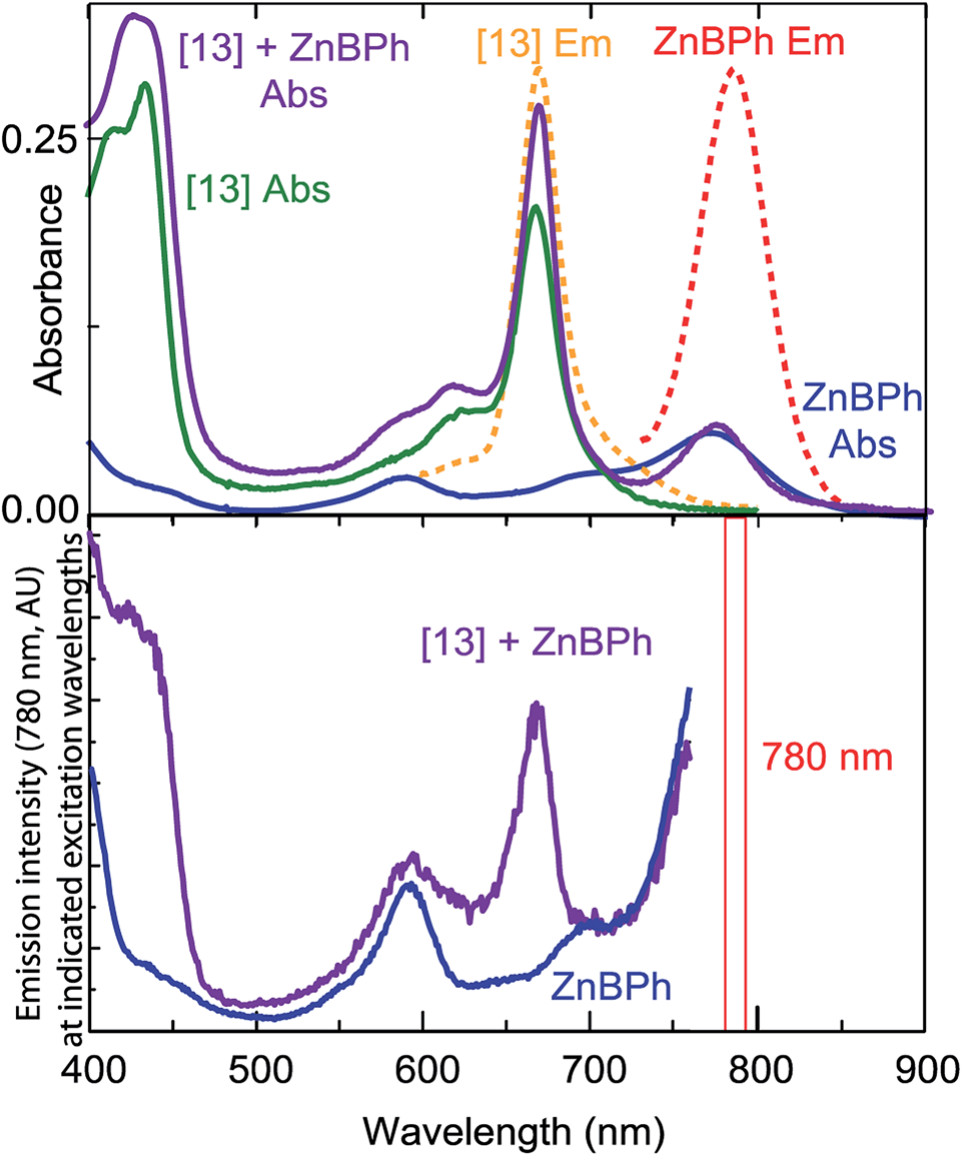
Top: Absorbance of maquette binding two different Zn tetrapyrroles, (**13**) and ZnBPh, independently (green or blue) and together (purple). Fluorescence emission profiles of the separate bound pigments are shown as orange or red dotted lines upon excitation at 590 or 720 nm. Bottom: Excitation spectrum for 780 nm emission from a maquette with both tetrapyrroles (purple) shows an absorbance at 670 nm not present when only ZnBPh is bound (blue) indicating energy transfer from (**13**) to ZnBPh.

When two different Zn tetrapyrroles are bound in a single maquette, energy transfer over the 2 nm center-to-center separations is generally evident, as long as there is a reasonable overlap of the emission of one chromophore with the absorbance of the other, such as the combination of tetrapyrroles (**11**) and (**12**). Fig. 8 illustrates another combination in which tetrapyrrole (**13**) with absorbance and emission near 660 nm (green and dashed orange lines, top of Fig. 8) overlaps moderately well with the absorbance of Zn bacteriopheophorbide (ZnBPh, blue line, top of Fig. 8). ZnBPh binding alone in a similar maquette has been reported.^13^ The maquette with both chromophores shows a compound absorbance spectrum (purple). Monitoring the emission from ZnBPh (bottom Fig. 8) at 780 nm, where there is very little emission from (**13**), shows a clear peak due to the absorbance of (**13**), revealing energy transfer between the tetrapyrroles.

## Discussion

Both photosynthetic light energy and electron transfer between tetrapyrrole pigments are conspicuously distance dependent. Thus successful operation of both natural and artificial light harvesting and light-activated redox proteins depends on secure anchoring of the appropriate tetrapyrroles in the proper location in the protein frames. Indeed, natural photosystems engineer a conspicuous gap between the antenna pigments on the one hand, and the more closely positioned charge separating reaction-center pigments on the other, and so avoid unproductive electron-transfer reactions with the antenna system.^19^

Just how natural Mg and Zn^20^ chlorophyll (Chl) and bacteriochlorophyll (BChl) cofactors are loaded *in vivo* into specific sites in the natural light harvesting systems has not been resolved.^21^ It is clear that a molecular chaperone is needed to enable light harvesting complex (LHC) proteins to insert properly into thylakoid membranes^22^ and that the protein folding requires the presence of the Chl cofactors. It is not clear how the Chl cofactor is managed before protein binding and how the potential threat of free Chl photo-oxidative reactions is avoided. Chl can be reconstituted into natural LHC proteins outside the cell, but this requires the assistance of detergent. In these systems, long tailed esterifying alcohols on the BChls and the carboxyl group on the mesoporphyrin ring are important structural requirements for binding.^4^ Many studies with artificial proteins also rely on detergent to solubilize light active tetrapyrrole cofactors and/or the proteins themselves.^23–26^ These systems are clarifying what factors determine the binding specificity and different protein-metal coordination preferences of detergent solubilized Chl a, b and c.^27^

Artificial light-harvesting proteins offer a wider engineering freedom of design compared to natural proteins. Furthermore, the protein maquettes described here, which have no significant sequence similarity to natural proteins, have relatively simple sequences and structures that allow the roles of individual amino acids and cofactor substituents to be more easily isolated to resolve general engineering principles. The conformationally rigid protein scaffolds of maquettes have interior binding sites that are sufficiently malleable to accommodate structurally diverse tetrapyrroles through variety of protein–cofactor interactions both inside and outside the cell.^5,8,9,11–13^ Multi-cofactor binding maquettes demonstrate light absorption, energy transfer and light-induced charge separation.^7,28,29^ Furthermore, because these proteins are designed from first principles of protein folding, they can be extremely stable, tolerating boiling temperatures in some cases; as seen in Fig. 7, cofactor binding generally increases the thermal stability of maquettes. These properties make the maquette platform attractive for engineering light activated energy converting systems outside of the cell as a biomaterial, and inside the cell, when integrated into natural bioenergetic pathways. Indeed, our ability to create water-soluble light-harvesting and charge-separating maquettes should allow us to design energy harvesting systems that work directly in the cytoplasm, free of cell membranes.

Previous work with water-soluble artificial proteins^6,11,13,30,31^ recognized that the water insolubility of natural Chls and BChls and other light active tetrapyrroles impedes binding. Often, water-soluble derivatives of Chl and BChl removing the hydrophobic tail were used instead.^13,30,31^ While increasing cofactor water solubility reduces aggregation in water, cofactor hydrophobicity is nevertheless useful for tight cofactor binding. The hydrophobic effect for binding in the non-polar protein interior is estimated at 2.4 kcal nm^–2^.^6,32^ Experimentally, we distinguish the energetic effect of hydrophobic portioning of a cofactor into a maquette interior from the effect of axial tetrapyrrole metal ligation by comparing maquette designs that are histidine-free from those with independent histidines orientated towards the bundle core ((**1**) *vs.* (**2**) *vs.* (**3**) in Fig. 2). Maquette tetrapyrrole binding occurs *via* a step-wise partitioning of the cofactor into the hydrophobic bundle interior, followed by histidine ligation.^33^ Clearly, for successful incorporation of light active tetrapyrrole cofactors into an artificial water-soluble protein frame, a balance must be struck between the hydrophobic and hydrophilic character of tetrapyrrole ring substituents. The work reported here was initiated to systematically define what makes a successful balance in order to provide a guide for cofactor construction and customization for a range of artificial light-harvesting protein functions.

*meso*-Substitutions that are either all charged/polar (**8**) or all non-polar (**3**) represent extremes of the hydrophobic/hydrophilic spectrum that fall prey to far too weak partitioning into the hydrophobic protein interior or aggregation in aqueous solution (Fig. 4). Iron tetrapyrrole self-assembly to bis-histidine sites displays a similar pattern.^33^ A charged/polar *meso*-substituent on one edge of the tetrapyrrole appears to offer the best compromise (**4**); this may be because hydrophobic burial of the other three edges may maximize hydrophobic effect forces. Adding charged groups to other faces ((**5**), (**6**), (**7**)) may well be forcing energetically unfavorable protein adjustments to expose these charges to the aqueous phase, or the energetic penalty of protonation/deprotonation of the charged group to neutrality.

Porphyrins with a single charged and non-polar substituent on opposite side of the tetrapyrrole perimeter without any other peripheral groups recapitulate the balance seen in the best tetra-substituted porphyrins, although binding may be slowed because of porphyrin stacking issues that can be present in aqueous solution (*e.g.* (**9**)). Increasing the number of charge groups on one edge (*e.g.* (**10**)) can clearly speed binding (Fig. S1†). Note that Zn protoporphyrin IX, an early successful cofactor choice for light activated water-soluble maquettes,^11^ falls into this category, with two charged propionates on one edge and non-polar methyl and vinyl groups on the other edges. A similar, even balance between a charged group and non-polar groups on opposite tetrapyrrole edges applies to successful chlorin self-assembly as well (Fig. 6). The pattern that emerges for rapid and tight self association of light active Zn tetrapyrroles to buried histidines in water soluble helical bundle proteins is to place one or more charge groups on one edge of the tetrapyrrole perimeter, hydrophobic groups up to ∼8 Å long on the opposite edge, with other non polar groups tolerated on the other two edges. Groups that disrupt pi stacking of tetrapyrroles in aqueous solution are also helpful. This pattern for single His ligated Zn tetrapyrrole binding is consistent with and extends the pattern previously described for a range of bis-His ligated Fe tetrapyrroles.^33^ Furthermore, this pattern appears generalizable not just to Fe *vs.* Zn tetrapyrrole cofactors, but to a range of different 4 helix bundle maquettes with different helical threading, loop length, helical charge and His positions (additional sequences and binding affinities in ESI†).

## Conclusions

This amphiphilic-cofactor binding model allows us to screen and choose among the variety of synthetic chlorins and bacteriochlorins with diverse spectroscopic and redox properties that have been synthesized so far.^34^ It also provides a crucial focus for synthesizing new chlorins to construct light-harvesting maquettes for absorbing customized wavelengths of solar radiation. Because maquettes can be constructed with histidine ligating sites of different binding affinities within the same protein, we have been able to sequentially load different cofactors to different sites within the same maquette. Thus we should be able to exploit differences in the binding affinities of porphyrins, chlorins and pheophorbides to engineer maquette systems accommodating multiple cofactors with diverse spectroscopic properties analogous to natural protein systems of photosynthesis.

Maquette modularity, self-assembly and robust adaptability to changes in the pattern of exterior amino acids is now being explored in the construction of nano and meso scale architectures for solar light capture.^35^ Preliminary results also show that with certain designs, not only hemes^8^ but natural chlorophyll and bilin tetrapyrroles self-assemble into maquettes inside the cell. However, further work is needed to resolve which protein binding site properties interact most favorably with natural or modified tetrapyrroles to optimize *in vivo* self-assembly, so that we can begin to tap into and divert energy flow of cellular bioenergetic systems towards a new class of light driven fuel production.

## Experimental

### Maquette expression

Codon optimized synthetic genes were obtained from DNA2.0 in PJ414 vector. The protein was expressed with a histidine tag in *E. coli* BL21 (DE3) cells for 5 hours at 37 °C, after induction with isopropyl-thiogalactopyranoside (IPTG) (0.5 mM).^10^ The cells were harvested by centrifugation, resuspended in KH_2_PO_4_ buffer with octylthioglucoside (1%), and lysed by sonication with a micro-tip attachment. Lysate was centrifuged at 25 000*g* for 25 minutes, with supernatant applied to a Ni nitrilotriacetic acid superflow resin (Qiagen) on an Akta FPLC. The His-tag was cleaved by recombinant tobacco etch virus N1a protease overnight, and final purification was *via* Waters reverse-phase HPLC. Molecular weight was assayed by either MALDI or ESI mass spectrometry.^7^

### Porphyrins

Zn(ii) 5,10,15,20-tetra(4-sulphonatophenyl)porphyrin (**1**), Zn(ii) 5,10,15-tri(4-sulphonatophenyl)-5-phenylporphyrin (**2**) and Zn(ii) 5,10,15,20-tetraphenylporphyrin (**3**) were purchased from Frontier Scientific, all other Zn porphyrins with different substituent groups were synthesized (for synthesis and characterization see ESI†) and will be referred to with the following numbers in the manuscript, Zn(ii) 5-4-(carboxyphenyl)-10,15,20-triphenylporphyrin (**4**), Zn(ii) 5,15-di(4-carboxyphenyl)-10,20-diphenylporphyrin (**5**), Zn(ii) 5,10-di(4-carboxyphenyl)-15,20-diphenylporphyrin (**6**), Zn(ii) 5,10,15-tri(4-carboxyphenyl)-5-phenylporphyrin (**7**), Zn(ii) 5,10,15,20-tetra(4-carboxyphenyl)porphyrin (**8**), Zn(ii) 5-phenyl-15-(*p*-carboxyphenyl)porphyrin (**9**) and Zn(ii) 5-phenyl-15-(*p*-carboxyphenyl)porphyrin with first generation Newkome dendrimer (**10**).

### Chlorins

Amphiphilic chlorins 15-(4-carboxyphenyl)-17,18-dihydro-18,18-dimethyl-5-*p*-tolylporphyrin (**11**) and Zn(ii) 15-(4-carboxyphenyl)-17,18-dihydro-18,18-dimethyl-3-(phenylethynyl)porphyrin (**12**) were synthesized with a *de novo* method that enables introduction of substituents at desired sites about the perimeter of the macrocycle.^36^ Acid-promoted condensation of the Eastern and Western halves is followed by metal-mediated oxidative cyclization. The selective introduction of substituents relies on (i) the use of substituted precursors (Eastern and Western halves); or (ii) bromination of the chlorin macrocycle followed by Pd-mediated coupling reaction. Both strategies were used in the synthesis of target chlorins. Pheophorbide a is purchased from Frontier scientific and Zn is inserted by refluxing 5 molar equivalents of ZnCl_2_ in methanol as previously described.^37^ Bacteriochlorophyll was extracted from *Rb. sphaeroides*, then demetalated, phytyl chain cleaved before Zn insertion to form Zn bacteriochlorophyllide.^37^

### UV/visible and circular dichroism (CD) spectroscopy

Protein solutions were prepared in CHES buffer (20 mM, 150 mM KCl, pH 9.0). Binding was monitored by UV/Vis Soret band absorbance on a Varian Cary-50 spectrophotometer at room temperature in a 1 cm path quartz cuvette. Secondary structure was monitored by CD spectroscopy (Aviv Model 410) at 25 °C with a 1 mm path quartz cuvette. Thermal denaturation was followed by monitoring the ellipticity at 222 nm every 5 °C after 15 minutes of equilibration. Melting temperatures were calculated using a Boltzmann equation with one term for each observed transition.

### Cofactor binding affinity

Pigment stock solutions were weighed out and solubilized in dimethylsulfoxide to give concentrations of 1 mM Zn porphyrins, 1 mM chlorin (**11**), 500 μM chlorin (**12**), and 2 mM chlorin (**13**). Binding of Zn porphyrins shift the Soret peak from 422 nm to 432 nm. Binding of chlorins shift the Soret band from 415 nm to 423 nm for (**12**), 411 nm to 425 nm for (**13**), and 409 nm to 417 nm for (**11**). Dissociation constants (*K*_d_ values) were determined as follows. To 1 ml of protein solution at 25 °C, 1 μl additions of 0.2 (porphyrin) or 0.1 (chlorin) equivalent aliquots were added successively to obtain a spectrum from 300 to 700 nm. At 1 equivalent of cofactor the DMSO concentration is 1%. Titrations typically ended at 4 equivalents of cofactor. The matrix of spectral absorbance from 300 to 700 nm for each of the additions of cofactor was subjected to singular value decomposition^16^ using a customized Mathematica program, revealing two dominant singular values, as expected for a system with two spectral species, one bound and one unbound. The amplitudes of the first two principle components as a function of total pigment added fit well to a mathematical model of a single dissociation constant for each protein binding site to generate fit spectral extinction coefficients at each wavelength for the unbound and bound pigment spectra as well as values and error estimates for the dissociation constants.

### Stopped-flow spectroscopy

Tetrapyrrole and protein were added to separate syringes of an OLIS RSM 1000 stopped flow spectrophotometer, which takes a full visible spectrum every millisecond through a 2 cm flow cell after rapid (∼2 ms deadtime) mixing. Temperature was controlled with a Fischer-Scientific IsoTemp 3031 water bath. Individual wavelengths were selected for further kinetic analysis. All experiments were performed in triplicate, and the data were averaged together for further analysis.
