# Supplementary material for: Design and engineering of water-soluble light-harvesting protein maquettes
Source: Chem Sci. 2016 Aug 17;8(1):316–24. doi: 10.1039/c6sc02417c (PMC5330312; doi:10.1039/c6sc02417c)
Supplement: Supplementary file 1 [file SC-008-c6sc02417c-s001.pdf]

Supporting Information

Title: Design and engineering of water-soluble light-harvesting protein maquettes

*Goutham Kodali, Joshua A. Mancini, Lee A. Solomon, Tatiana Esipova, Nicholas Roach, Christopher J. Hobbs, Pawel Wagner, Olga A. Mass, Kunche Aravindu, Jonathan E. Barnsley, Keith C. Gordon, David L. Officer, P. Leslie Dutton and Christopher C. Moser**

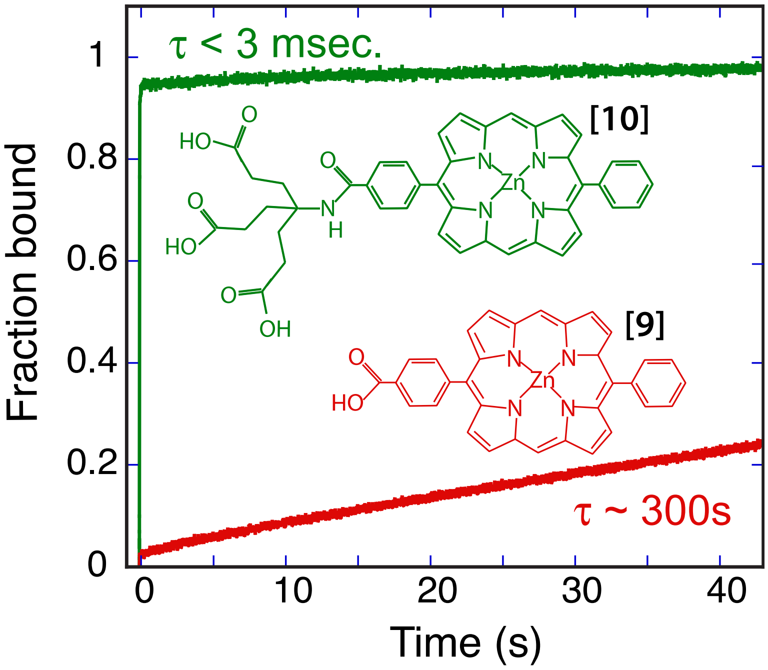

Figure S1: Proliferating carboxylic groups on the 15-porphyrin substituent leads to faster binding as seen by stopped flow measurements. Conditions: 2 μM of protein maquette in 20 mM CHES, 150 mM KCl mixed with one equivalent of porphyrin, either 5-phenyl-15-carboxy phenylporphyrin [9] (red) or 5-phenyl-15-Newkome phenylporphyrin [10] (green). Normalized absorbance increase with time in seconds.

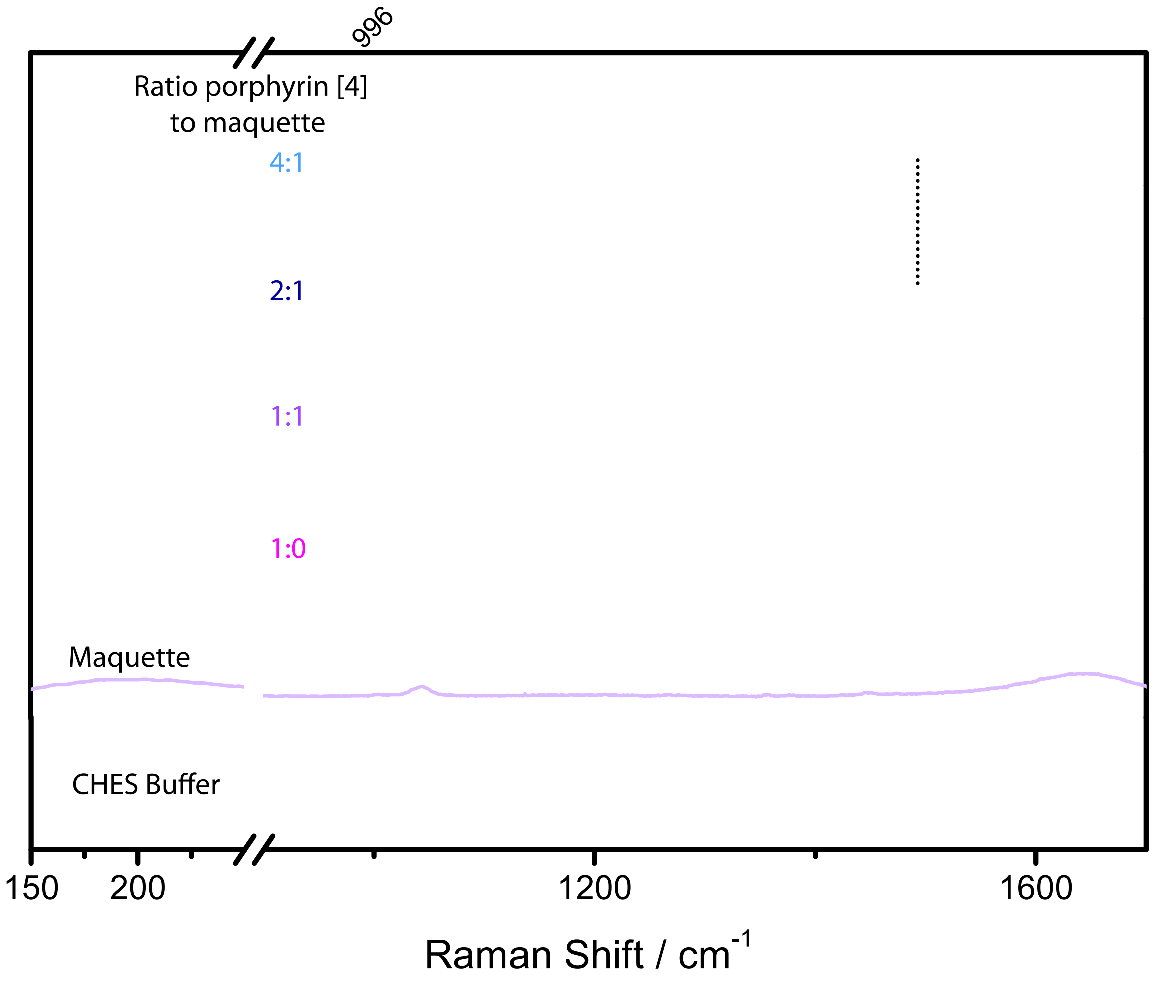

Figure S2: Resonance Raman spectra for porphyrin **[4]** excited at 448 nm at stoichiometric ratios of [**4**] to 2-His maquette: 1:0 (magenta), 1:1 (purple), 2:1 (one porphyrin per His site, dark blue), and 4:1 (cyan). CHES buffer alone (black) and maquette alone in buffer (light purple) show little Raman activity. Peaks near 1000 cm^{-1} show intensity changes of the 995 cm^{-1} shoulder and blue shifts as maquette is added. The intensity of band 1420 cm^{-1} consistently decreases on addition of maquette.

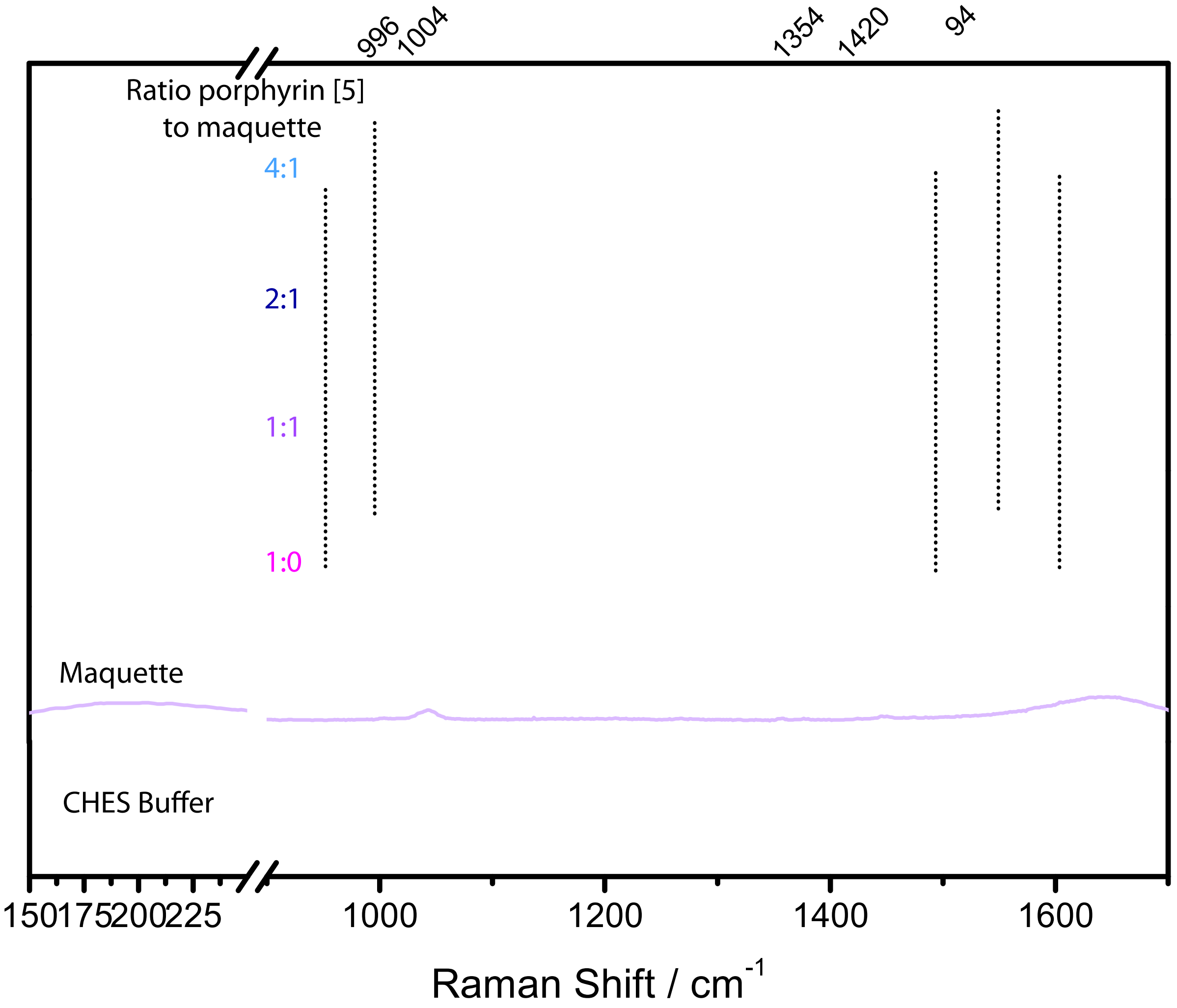

Figure S3: Resonance Raman spectra for porphyrin **[5]** excited at 448 nm at stoichiometric ratios of [**5**] to 2-His maquette: 1:0 (magenta), 1:1 (purple), 2:1 (one porphyrin per His site, dark blue), and 4:1 (cyan). Peaks near 1000 cm^{-1} show intensity changes of the 995 cm^{-1} shoulder as maquette is added. The intensity of band 1420 cm^{-1} consistently decreases on addition of maquette.

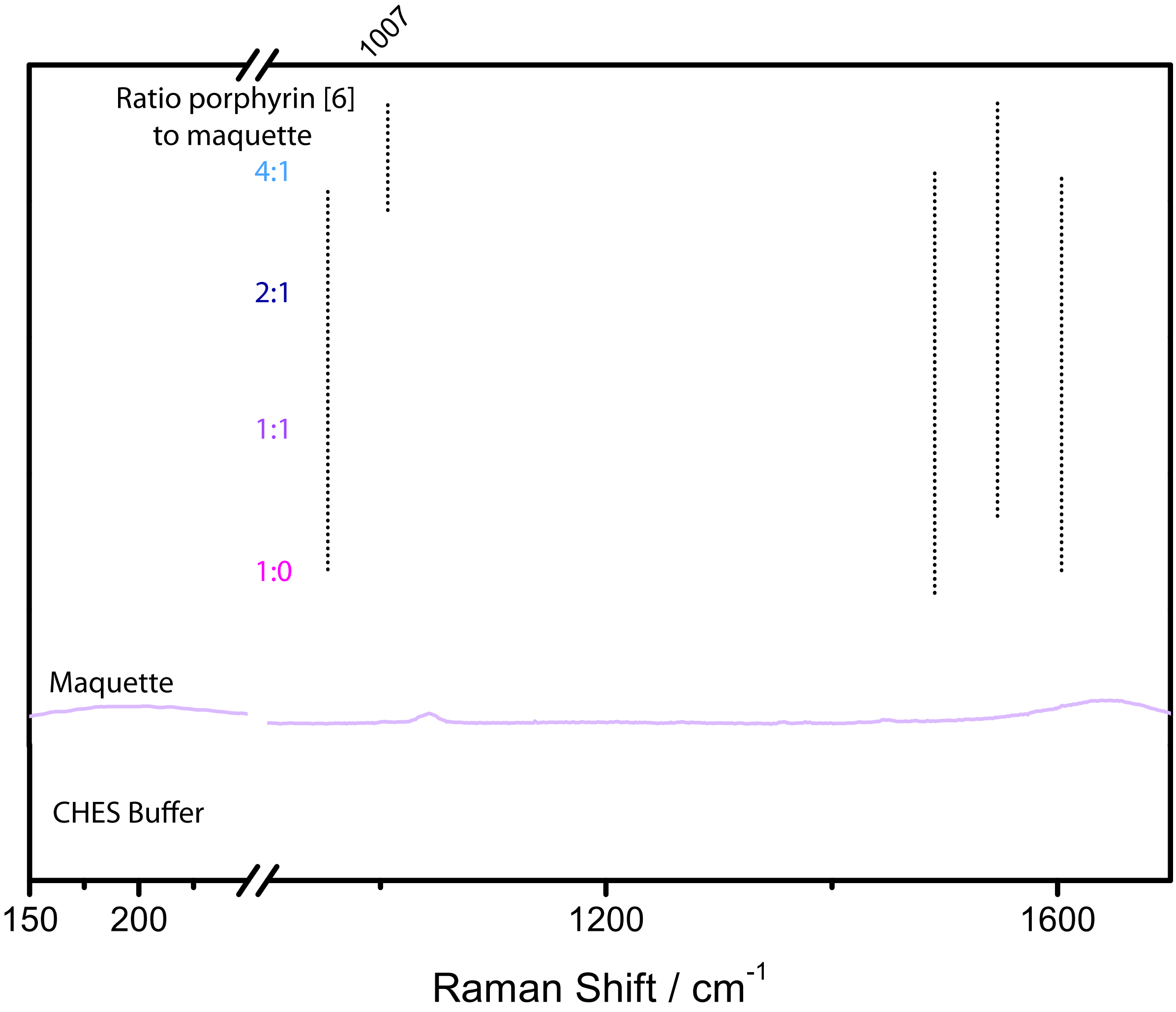

Figure S4: Resonance Raman spectra for porphyrin **[6]** excited at 448 nm at stoichiometric ratios of [**6**] to 2-His maquette: 1:0 (magenta), 1:1 (purple), 2:1 (one porphyrin per His site, dark blue), and 4:1 (cyan). Peaks near 1000 cm^{-1} show intensity changes of the 995 cm^{-1} shoulder as maquette is added. The intensity of band 1420 cm^{-1} consistently decreases on addition of maquette.

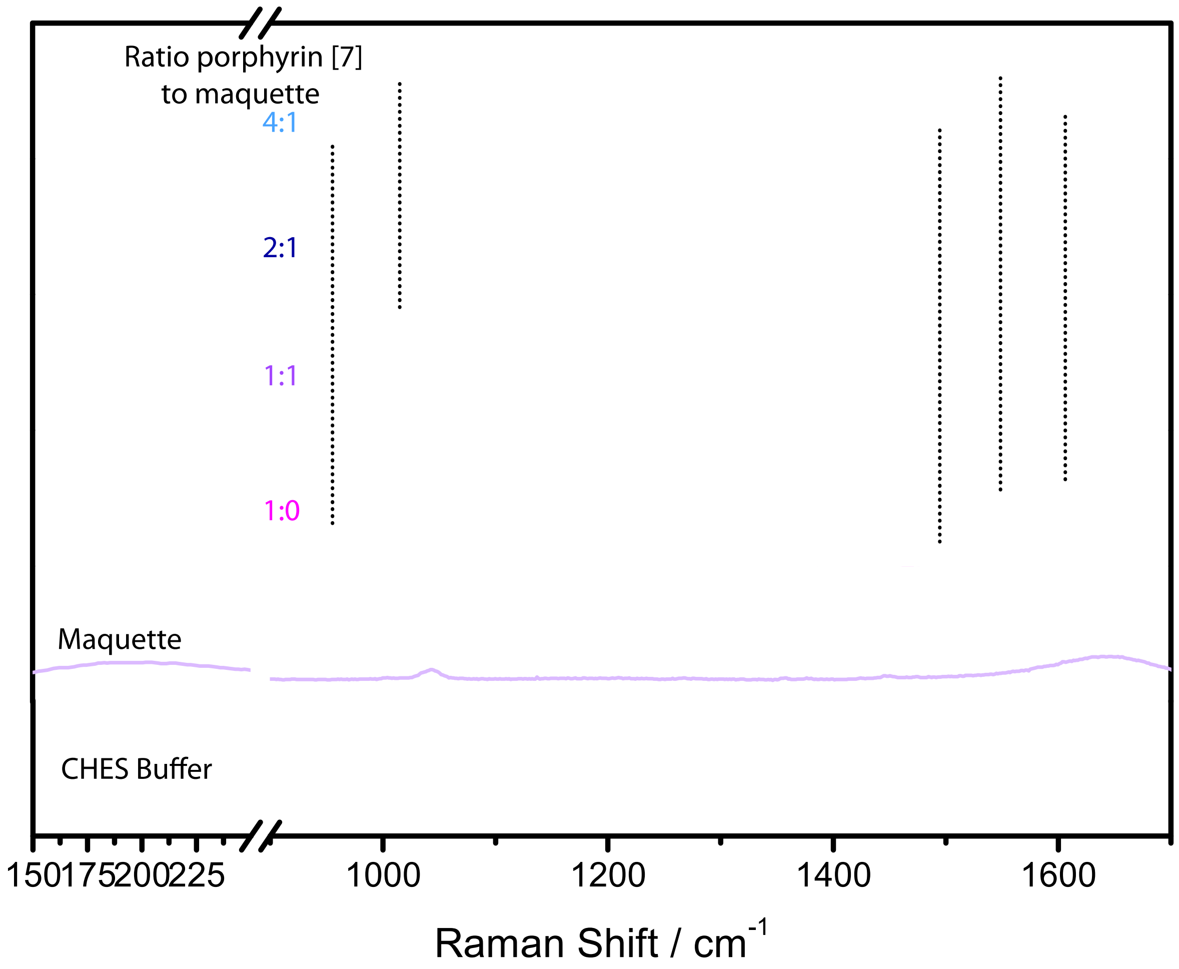

Figure S5: Resonance Raman spectra for porphyrin **[7]** excited at 448 nm at stoichiometric ratios of [**7**] to 2-His maquette: 1:0 (magenta), 1:1 (purple), 2:1 (one porphyrin per His site, dark blue), and 4:1 (cyan). Peaks near 1000 cm^{-1} show intensity changes of the 995 cm^{-1} shoulder as maquette is added. The intensity of band 1420 cm^{-1} consistently decreases on addition of maquette.

All porphyrin synthesis: All reagents and solvents were commercially available and used without additional purification, unless stated differently. Column chromatography was performed on Selecto™ silica gel (Fisher Scientific). NMR spectra were recorded on a Bruker Avance 400 spectrometer or Bruker DPX-400 spectrometer. The following abbreviations are used: s = singlet, d = doublet, dd = doublets of doublets, ddd = doublets of doublets of doublets, m = multiplet. All coupling constants J were measured in hertz (Hz). Chemical shifts are reported in parts per million (ppm). Tetramethylsilane was used as the internal reference. Mass spectra were recorded on a Polaris Q or Hewlett Packard 5973 mass spectrometer. MALDI-TOF mass spectra were recorded on a MALDI-TOF Bruker Daltonics Microflex LRF instrument, using α-cyano-4-hydroxycinnamic acid (CCA) as a matrix (positive-ion mode). Samples were prepared by mixing solution of the analyte compound in THF or MeOH (10 μL, ~1 mM) with solution of the matrix (100 μL, 10 mg/ml, 0.053 M) in THF. The sample, approximately 1 μL, was then deposited on the probe tip, dried and analyzed. HRMS spectra were recorded on an ESI (electro-spray ionization) Thermo

Scientific LTQ Orbitrap XL instrument using direct injection of a sample, consisting of ~10 μM solution of the analyte compound in MeOH or THF/MeOH mixture (1:1). UV-vis spectra were recorded in dichloromethane unless stated differently on Shimadzu UV-1800 spectrophotometer.

General arylporphyrin acid preparation: Investigated arylporphyrin acids were isolated from statistical mixtures prepared from pyrrole and aldehydes bearing the required substituents. The yield and spectral data of the pure, isolated porphyrins is given below each procedure with reference to a previous synthesis if applicable. Carboxylic acids were obtained from the corresponding methyl esters by hydrolysis following chromatographic separation and zinc insertion as detailed below. Assessment of the purity of the porphyrin esters was performed by ^{1}H NMR spectroscopy but this was not applicable to the ABAB isomers because of complete spectral overlap with the AABB isomers. Where necessary, HPLC analysis, using dichloromethane as the mobile phase, was used to assess the degree of separation and purity. Yields were calculated based on the conversion of pyrrole.

General zinc insertion conditions: To a solution of the porphyrin (0.025 mmol) in dichloromethane, zinc acetate dihydrate (11 mg, 0.049 mmol) dissolved in methanol (1 mL) was added. The reaction was monitored by TLC using dichloromethane as mobile phase and was complete within two hours. The solution was concentrated under reduced pressure and methanol was added to induce precipitation of the zinc porphyrin, which was then collected by filtration. Conversion to the zinc complex was quantitative in each case.

General ester hydrolysis conditions: Zinc complex of a porphyrin benzoate (0.039 mmol) was dissolved in tetrahydrofuran (10 mL). A mixture of potassium hydroxide (116 mg, 2.08 mmol), water (0.9 mL) and methanol (9 mL) was added. The solution was heated at reflux for 3 hours and then allowed to cool down to room temperature. Water (10 mL) was added and the organic solvents were evaporated under reduced pressure. The solution was acidified with phosphoric acid (85%), which induced precipitation of the porphyrin. The porphyrin was compressed to a pellet by centrifugation at 2500 rpm for 10 minutes. The supernatant liquid was then removed with a pipette, water added and centrifugation repeated. After four rounds of centrifugation, the porphyrin was dried under a flow of nitrogen and then in a vacuum oven at 50°C.

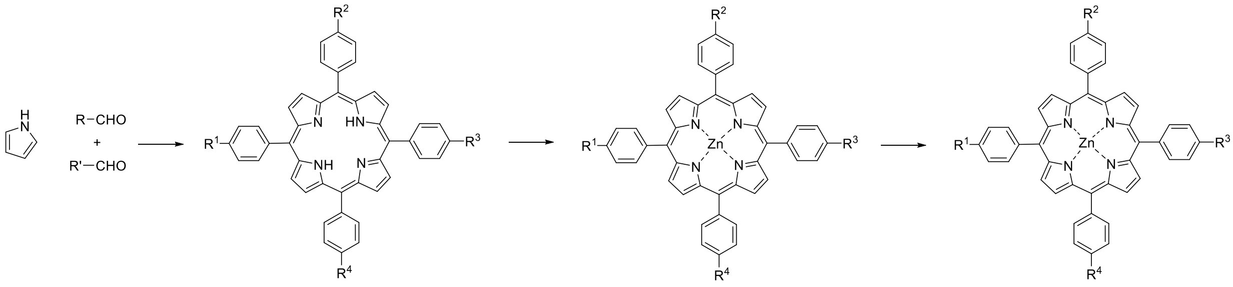

a: R1 = R2 = R3 = R4 = H
b: R1 = R2 = R3 = H; R4 = COOMe (H)
c: R1 = R2 = H; R3 = R4 = COOMe (H)
d: R1 = R3 = H; R2 = R4 = COOMe (H)
e: R1 = H; R2 = R3 = R4 = COOMe (H)
f: R1 = R2 = R3 = R4 = COOMe (H)

[3]: R1 = R2 = R3 = R4 = H
[4]: R1 = R2 = R3 = H; R4 = COOH
[5]: R1 = R2 = H; R3 = R4 = COOH
[6]: R1 = R3 = H; R2 = R4 = COOH
[7]: R1 = H; R2 = R3 = R4 = COOH
[8]: R1 = R2 = R3 = R4 = COOH

Figure S6: 3 step synthesis of arylporphyrin acids

Preparation of Arylporphyrin acids [4]-[8]:

[4]: 5-(4-(Carboxyphenyl)-10,15,20-triphenylporphyrinato zinc (II) [1]

^{1}H NMR (400 MHz, DMSO-d$_6$): δ = 7.72 - 7.82 (m, 9H, ArH), 8.19 - 8.25 (m, 8H, ArH), 8.33 – 8.38 (m, 2H, ArH), 8.47 - 8.51 (m, 2H, ArH), 8.90 (d, *J* = 4.8 Hz, 2H, β pyr H), 8.96 (s, 4H, β pyr H), 8.98 (d, *J* = 4.8 Hz, 2H, β pyr H); λ_{max} (log ε): 408 (4.6), 428 (5.6), 561 (4.3), 601 (4.0); HRMS (ESI, M$^+$): found: 720.1501, requires for $C_{45}H_{28}N_4O_2Zn$: 720.1504.

[5]: 5,10-Di(4-carboxyphenyl)-15,20-diphenylporphyrinato zinc (II) (3d) [2]

^{1}H NMR (400 MHz, DMSO-d$_6$): δ = 7.75 - 7.85 (m, 6H, ArH), 8.14 - 8.21 (m, 8H, ArH), 8.30 (d, *J* = 7.5 Hz, 4H, ArH), 8.77 (d, *J* = 4.6 Hz, 4H, β pyr H), 8.79 (d, *J* = 4.6 Hz, 4H, β pyr H); λ_{max} (log ε): 408 (4.7), 429 (5.8), 561 (4.3), 601 (4.0); HRMS (ESI, M$^+$): found: 764.1412, requires for $C_{46}H_{28}N_4O_4Zn$: 764.1402.

[6]: 5,15-Di(4-carboxyphenyl)-10,20-diphenylporphyrinato zinc (II) [2]

^{1}H NMR (400 MHz, DMSO-d$_6$): δ = 7.76 - 7.85 (m, 6H, ArH), 8.16 - 8.22 (m, 4H, ArH), 8.28 (d, *J* = 8.2 Hz, 4H, ArH), 8.35 (d, *J* = 8.1 Hz, 4H, ArH), 8.77 (d, *J* = 4.8 Hz, 4H, β pyr H), 8.79 (d, *J* = 4.8 Hz, 4H, β pyr H); λ_{max} (log ε): 408 (4.6), 429 (5.7), 562 (4.3), 602 (4.1); HRMS (ESI, M$^+$): found: 764.1425, requires for $C_{46}H_{28}N_4O_4Zn$: 764.1402.

[7]: 5,10,15-Tri[4-carboxyphenyl)-5-phenylporphyrinato zinc (II) [2]

^{1}H NMR (400 MHz, DMSO-d$_6$): δ = 7.78 - 7.83 (m, 3H, ArH), 8.16 - 8.21 (m, 8H, ArH), 8.27 (d, *J* = 8.0 Hz, 6H, ArH), 8.35 (d, *J* = 8.0 Hz, 6H, ArH), 8.76 - 8.81 (m, 8H, β pyr H); λ_{max} (log ε): 409 (4.5), 429 (5.6), 562 (4.2), 602 (4.0); HRMS (ESI, M$^+$): found: 808.1302, requires for $C_{47}H_{28}N_4O_6Zn$: 808.1300.

[8]: 5,10,15,20-Tetra(4-carboxyphenyl)porphyrinato zinc (II) [3]

^{1}H NMR (400 MHz, DMSO-d$_6$): δ = 8.23 (d, *J* = 7.5 Hz, 8H, ArH), 8.32 (d, *J* = 7.5 Hz, 8H, ArH), 8.79 (s, 8H, β pyr H); λ_{max} (log ε): 410 (4.5), 430 (5.5), 565 (4.7), 605 (4.6).

Newkome porphyrin synthesis:

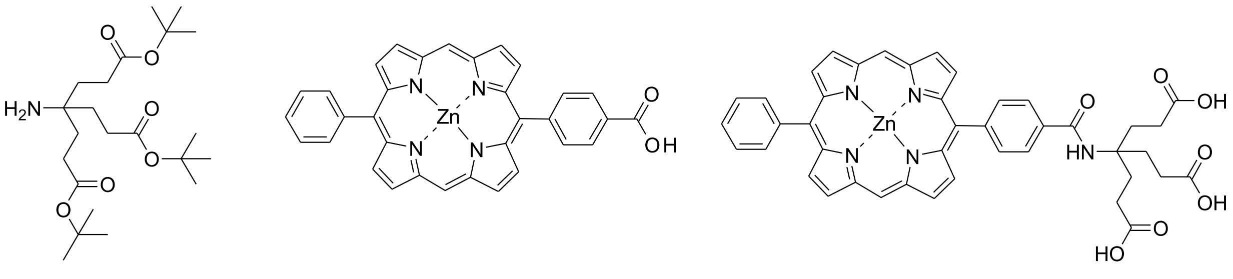

NwOtBu [9] [10]

Figure S7: Components of Newkome porphyrin synthesis.

NwOtBu was purchased from Frontier Scientific, Inc. (NTN1963, CAS 136586-99-7). 5-Phenyldipyrromethane and 5-(4-methoxycarbonylphenyl)dipyrromethane were prepared following the published methods [4].

1,9-bis(*N,N*-Dimethylaminomethyl)-5-(4-methoxycarbonylphenyl)dipyrromethane was prepared in accordance with Lindsey method [5]. To a solution of 5-(4-methoxycarbonylphenyl)dipyrromethane (0.9468 g, 2.9 mmol) in CH_2Cl_2 (50 mL), N,N-dimethylethyleneiminium iodide (Eschenmoser's salt) (1.3871 g, 6.4 mmol) was added. The reaction mixture was stirred for 1 h at room temperature, then diluted with CH_2Cl_2 (350 mL), and K_2CO_3 (10% aq., 350 mL) was added. The organic layer was separated, washed with

K_2CO_3 (10% aq., 3×350 mL), dried over Na_2SO_4, and concentrated in vacuum. The title compound was precipitated from CH_2Cl_2 upon addition of hexanes, collected by filtration and dried in vacuum. Yield: 0.8223 g, (62%), pale yellow crystalline powder. NMR ^1H (DMSO-d_6), δ, ppm: 2.07 (12H, s, -NC<u>H</u>$_3$), 3.26 (4H, d, ^3J=2.51 Hz, -C<u>H</u>N(CH_3)$_2$), 3.81 (3H, s, -OC<u>H</u>$_3$), 5.37 (1H, s, -C<u>H-</u>), 5.53 (1H, d, ^3J=2.8 Hz, β pyr H), 5.54 (1H, d, ^3J=2.8 Hz, β pyr H), 5.72 (1H, d, ^3J=2.8 Hz, β pyr H), 5.73 (1H, d, ^3J=2.8 Hz, β pyr H), 7.23 (2H, d, ^3J=8.3 Hz, ArH), 7.85 (2H, d, ^3J=8.3 Hz, ArH), 10.51 (2H, s, -N<u>H</u>); NMR ^{13}C ($CDCl_3$), δ, ppm: 44.2, 44.9, 52.0, 56.6, 107.1, 107.5, 107.6, 128.4, 128.6, 128.9, 129.0, 129.8, 131.70, 131.74, 147.65, 147.67, 167.0. HRMS (ESI-TOF) m/z: $[M+H]^+$ Calcd for $C_{23}H_{31}N_4O_2$ 395.24413; Found: 395.24368.

Zn(II) 5-phenyl-15-(*p*-methoxycarbonylphenyl)-porphyrin [5,6].
1,9-bis(*N*,*N*-Dimethylaminomethyl)-5-(4-methoxycarbonylphenyl)dipyrromethane (1.5 g, 3.83 mmol) and 5-phenyldipyrromethane (0.842 g, 3.83 mmol) were dissolved in methanol (380 mL), and the reaction mixture was purged with Ar. An excess of $Zn(OAc)_2 \cdot 2H_2O$ (8.4 g, 38.3 mmol) was added, and the reaction mixture was refluxed for 2 h, during which time the solution color turned deep red. After 2 h, the reaction mixture was cooled down to room temperature, DDQ (2.6 g, 11.5 mmol) was added, and the mixture was allowed to react overnight. The methanol was evaporated under reduced pressure, and the product was purified by column chromatography (silica gel, CH_2Cl_2). The fraction containing the target porphyrin (monitored by UV-vis spectroscopy) was collected, evaporated to dryness to give the title compound as a purple solid. Yield 0.24 g (11%). All characterizations correspond to that previously reported [7].

[9]: Zn(II) 5-phenyl-15-(*p*-carboxyphenyl)-porphyrin [7].
To a solution of Zn(II) 5-phenyl-15-(*p*-methoxycarbonylphenyl)-porphyrin (0.048 g, 0.082 mmol) in THF (25 ml), solid NaOH (0.020 g, 0.5 mmol), MeOH (0.025 ml) and water (0.025 ml) were added. The reaction mixture was stirred at r.t. until the precipitate was formed. The solvent was carefully decanted, the precipitate was re-dissolved in water (20 ml) and stirred overnight at r.t. in the dark. The solution was acidified with 10% HCl to pH 3-4. The red precipitate formed was centrifuged, washed with water 3 times and dried in vacuum. Yield 0.0434 g, 93%. All characterizations correspond to that previously reported [7].

[10]: Zn(II) $P(Ar)_2NwOH$.
To a solution of Zn(II) 5-phenyl-15-(*p*-carboxyphenyl)-porphyrin (0.0205 g, 0.036 mmol) in dry DMF (10 ml), HBTU (0.017 g, 0.045 mmol) was added, and the reaction mixture was stirred for 10 min at r.t. DIPEA (0.3 ml, 0.18 mmol) was added to the reaction mixture in one portion, followed by the addition of the solution of NwO*t*Bu (0.0187 g, 0.045 mmol) in dry DMF (2 ml). After the stirring the mixture in the dark for 48 h at r.t., MALDI analysis confirmed the completeness of the reaction. The reaction mixture was poured into ice-cold water (100 ml), and a few drops of conc. HCl were added. The purple precipitate formed was centrifuged, washed 3 times with water and dried in vacuum. The crude product was purified by column chromatography (silica gel, CH_2Cl_2/THF, 20:1) to give 0.03 g (88%) of $P(Ar)_2$NwO*t*Bu. MALDI-TOF (m/z), calcd for $C_{55}H_{59}N_5O_7Zn$: 965.37, found: 965.34 $[M]^+$.
$P(Ar)_2$NwO*t*Bu (0.03 g, 0.031 mmol) was dissolved in TFA (5 ml), and the reaction mixture was stirred for 2 h at r. t. TFA was removed in vacuum, the porphyrin was re-dissolved in MeOH (10 m), and the excess of $Zn(OAc)_2 \cdot 2H_2O$ (0.068 g, 0.31 mmol) was added. The reaction mixture was refluxed for 1 h until complete conversion of the free-base porphyrin into the corresponding Zn-complex (monitored by UV-Vis spectroscopy). MeOH was

removed in vacuum, and the solid was washed with water (pH 6-7, 5×20 ml) and dried in vacuum to give the title compound as a purple solid. Yield 0.02 g (81%). NMR ^{1}H (DMSO-d_6), δ, ppm: 2.10-2.19 (6H, m, -CH_2-), 2.29-2.37 (6H, m, -CH_2-), 7.78-7.85 (3H, m, ArH), 7.01-7.97 (1H, s, broad, -NH), 8.18-8.29 (6H, m, ArH), 8.95 (2H, d, ^3J=4.4 Hz, β pyr H), 8.97 (2H, d, ^3J=4.4 Hz, β pyr H), 9.51 (2H, d, ^3J=4.4 Hz, β pyr H), 9.52 (2H, d, ^3J=4.4 Hz, β pyr H), 10.39 (2H, s, *meso*-H), 12.19 (3H, s, broad, -COOH).MALDI-TOF (m/z), calcd for $C_{43}H_{35}N_5O_7Zn$: 797.18, found: 797.14 $[M]^+$.

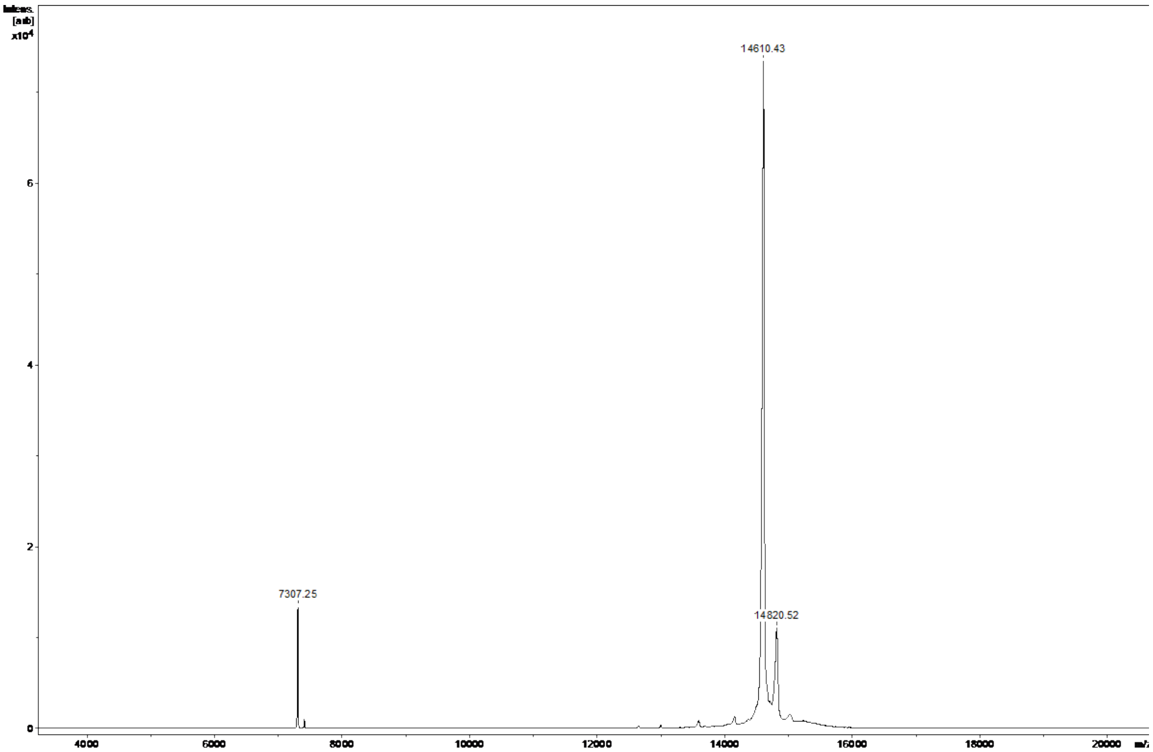

Figure S8: Mass spectrometry MALDI for 2-His maquette.

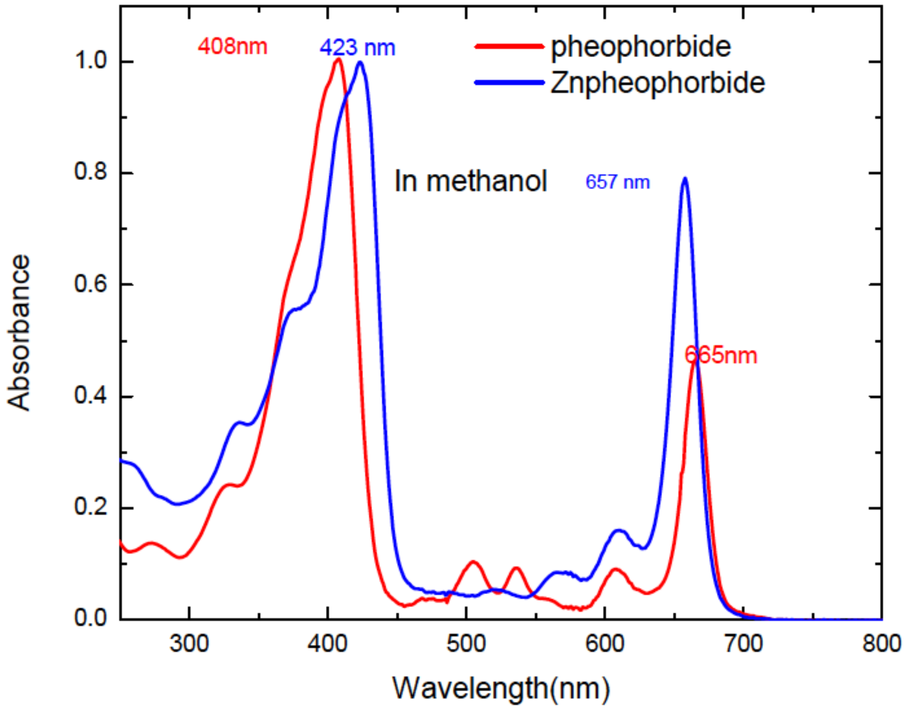

Figure S9: Absorbance spectra before and after Zn pheophorbide a metal insertion.

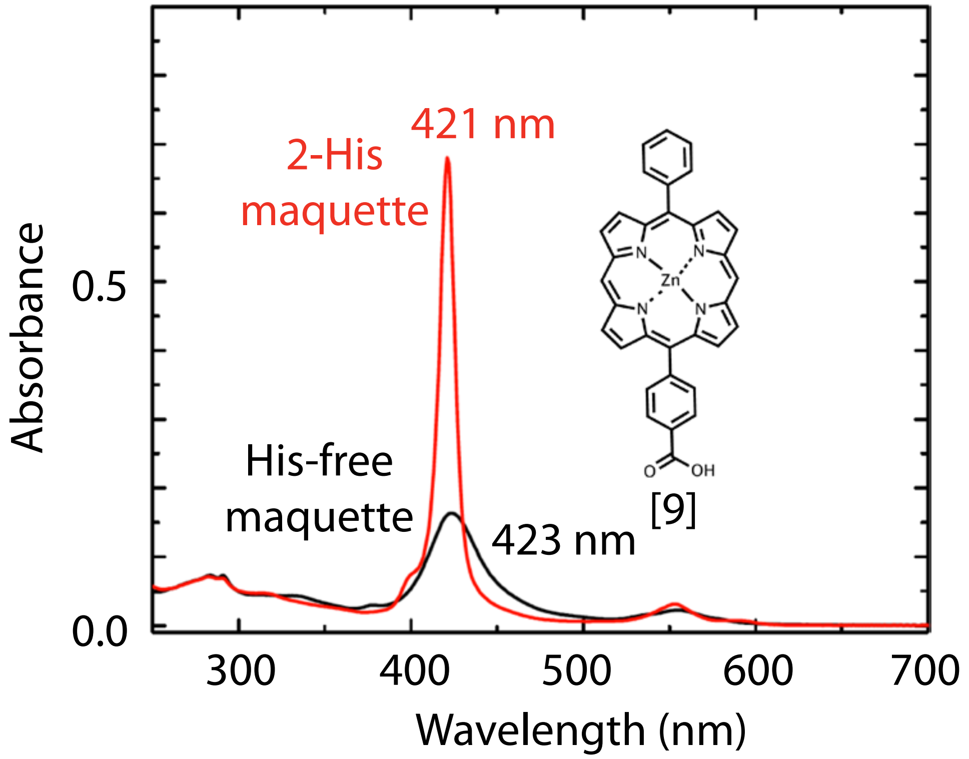

Figure S10: Absorbance spectra of porphyrin [9] in 2-His (red) and His-free (black) maquette

Supporting References:

(1) Harada, A.; Shiotsuki, K.; Fukushima, H.; Yamaguchi, H.; Kamachi, M.; *Inorg. Chem.*, **1995**, *34*, 1070.

(2) Koehorst, R. B. M.; Boschloo, G. K.; Savenije, T. J.; Goossens, A.; Schaafsma, T. J.; *J. Phys. Chem. B*, 2000, *104*, 2371.

(3) Verchere-Beaur, C.; Perree-Fauvet, M.; Gaudemer, A.; *J. Inorg. Biochem.*, 1990, *40*, 127.

(4) Laha, J. K.; Dhanalekshmi, S.; Taniguchi, M.; Ambroise, A.; Lindsey, J. S. *Org. Process Res. Dev.* 2003, *7*, 799.

(5) Fan, D. Z.; Taniguchi, M.; Yao, Z.; Dhanalekshmi, S.; Lindsey, J. S. *Tetrahedron* 2005, *61*, 10291.

(6) Clarke, O. J.; Boyle, R. W. *Tetrahedron Lett.* 1998, *39*, 7167.

(7) Zhang, N. N.; Feng, Y. Q.; Li, Y. C.; Peng, X.; Gu, C. Z.; Xue, X. D.; Yan, J. Y.; Chen, Q. L.; Li, X. G.; Zhang, B. *New Journal of Chemistry* 2013, *37*, 1134.
